# Supplementary material for: Magneto-transport evidence for strong topological insulator phase in ZrTe5
Source: Nat Commun. 2021 Nov 19;12:6758. doi: 10.1038/s41467-021-27119-5 (PMC8604917; doi:10.1038/s41467-021-27119-5)
Supplement: Supplementary file 1 — Supplementary Information [file 41467_2021_27119_MOESM1_ESM.pdf]

Supplementary Information: Magneto-transport evidence for strong topological insulator phase in $ZrTe_5$

Jingyue Wang,[1,2] Yuxuan Jiang,[3,4,*] Tianhao Zhao,[1] Zhiling Dun,[1] Anna L. Miettinen,[1] Xiaosong Wu,[2] Martin Mourigal,[1] Haidong Zhou,[5] Wei Pan,[6] Dmitry Smirnov,[4] and Zhigang Jiang[1,†]

[1]*School of Physics, Georgia Institute of Technology, Atlanta, Georgia 30332, USA*

[2]*State Key Laboratory for Artificial Microstructure and Mesoscopic Physics, Peking University, Beijing 100871, China*

[3]*School of Physics and Optoelectronics Engineering, Anhui University, Hefei, Anhui 230601, China*

[4]*National High Magnetic Field Laboratory, Tallahassee, Florida 32310, USA*

[5]*Department of Physics and Astronomy, University of Tennessee, Knoxville, Tennessee 37996, USA*

[6]*Quantum and Electronic Materials Department, Sandia National Laboratories, Livermore, California 94551, USA*

This file includes:

Supplementary Note 1. Additional sample characterization data

Supplementary Note 2. Landau level calculation and Zeeman effect

Supplementary Note 3. Four-fold splitting in the $n = 1$ LL

Supplementary Note 4. Magneto-resistance and magneto-thermopower calculation

Supplementary Figure 1. R versus T curve of our $ZrTe_5$ sample

Supplementary Figure 2. Robust peak positions in ΔR_{xx} independent of background subtraction methods

Supplementary Figure 3. Hall resistance and Nernst signal measured at low temperatures

Supplementary Figure 4. Zoom-in view of the Hall resistance data near zero magnetic field

Supplementary Figure 5. Temperature-dependent MR measurements

Supplementary Figure 6. Two possible assignments to the four-fold splitting in the $n = 1$ LL

1. Additional sample characterization data

The $ZrTe_5$ single crystals studied in this work exhibit a metallic behavior at low temperatures and a Lifshitz-transition-like resistance (R) peak at $T = 133$ K, as shown in the R versus T curve of Fig. 1. The inset to Fig. 1 illustrates the atomic structure of $ZrTe_5$. The resistance R, as well as the magneto-resistance MR in the main text, are measured via sending a current along the a direction, using the standard lock-in method.

In analyzing the quantum oscillation data in Fig. 2 of the main text, we obtain ΔR_{xx} by subtracting a smooth background (we choose R_{xx}(50K) as the background) from R_{xx}, $\Delta R_{xx} = R_{xx}(2.3\text{K}) - R_{xx}(50\text{K})$. Alternatively, we can choose a smooth high-order polynomial background, such as that shown in Fig. 2. We find that the peak positions in ΔR_{xx} are robust against the choice of the background, suggesting that the period of quantum oscillations is not shifted due to the background subtraction. The splitting of the $n = 1$ peak in MR seems more obvious when using the high-order polynomial background.

Figure 3 shows the Hall resistance and Nernst signal measured at low temperatures. We note that: (1) The negative slope of the Hall data at low magnetic fields indicates the dominant carriers are electrons in our samples, consistent with the sign of the thermopower

signal in Fig. 3 of the main text. (2) At high magnetic fields ($B > 18$ T), the Hall resistance decreases considerably and the slope becomes positive, which might suggest the emergence of a hole type of carriers, as speculated in the main text in the context with Fig. 3(a) at a similar magnetic field.

Figure 4 shows a zoom-in view of the Hall resistance data near zero magnetic field. We note that the nonlinear behavior in the data is very similar to that reported in Ref. [1], and it cannot be explained using a two-carrier (or multiple-carrier) model. Instead, such behavior can be decomposed into a normal Hall effect (linear in B-field) and an anomalous Hall effect (near zero field). The latter has been studied extensively in Refs. [2, 3], while anomalous thermopower effect has been reported in Ref. [4].

Figure 5 shows a more detailed temperature-dependent MR measurement on another device of 205 nm thick fabricated from $ZrTe_5$ samples of the same crystal growth. To obtain the oscillatory component ΔR, we subtract a smooth background from the MR data at different temperatures using a two-step procedure: (1) First, we subtract the 50 K high temperature background as described above; (2) Next, we subtract a polynomial background. The results after background subtraction are shown in Fig. 5(b). The oscillation amplitude of the $n = 2$ peak as a function of temperature is plotted in Fig. 5(c), which follows the Lifshitz-Kosevich (LK) formula

$$\frac{\Delta R}{R(0\mathrm{T})} \propto \frac{2\pi^2 k_B m^* T/\hbar eB}{\sinh(2\pi^2 k_B m^* T/\hbar eB)},$$

where k_B is the Boltzmann constant, m^* is the effective mass, $\hbar$ is the reduced Planck's constant, and e is the electron charge. We can extract an effective mass of $m^* = 0.045 m_e$ for the $n = 2$ LL, which is slightly larger than the values reported in the literature due to the higher carrier density in our samples.

To estimate the carrier density n_e, we consider the Fermi surface of $ZrTe_5$ as an ellipsoid [5] with $k_c : k_a = 1.30$ and $k_b : k_a = 7.93$, where $k_{a,b,c}$ are the principal axes. Following the Onsager relation, the quantum oscillation frequency $F = \hbar S_e / 2\pi e$, where $S_e = \pi k_a k_c$ is the Fermi surface area perpendicular to the magnetic field, and using average $F = 4$ T from Fig. 3(c) of the main text, we arrive at $n_e = k_a k_b k_c / 3\pi^2 \approx 3.2 \times 10^{17}$ cm^{-3}.

2. Landau level calculation and Zeeman effect

In this section, we show how the band inversion parameter $\mathscr{B}$ can split the LLs and the role of the Zeeman effect in LL splitting. To simplify the discussion, we use the following bases: $(|+,\uparrow\rangle, |-,\uparrow\rangle, |+,\downarrow\rangle, |-,\downarrow\rangle)$, where the orbitals and spins are denoted by $\pm$ and $\uparrow,\downarrow$, respectively. The effective Hamiltonian then reads

$$H(\mathbf{k}) = \begin{pmatrix} L(\mathbf{k}) & Ak_+ & 0 & A_z k_z \\ Ak_- & -L(\mathbf{k}) & A_z k_z & 0 \\ 0 & A_z k_z & L(\mathbf{k}) & -Ak_- \\ A_z k_z & 0 & -Ak_+ & -L(\mathbf{k}) \end{pmatrix}, \tag{1}$$

with $k_\pm = k_x \pm i k_y$, $L(\mathbf{k}) = M - \mathscr{B}_\perp(k_x^2 + k_y^2) - \mathscr{B}_z k_z^2$, and $A_{(z)} = \hbar v_{F(z)}$. We assume that the material parameters are isotropic in the $x - y$ plane but different along the z direction (that is, the layer stacking b direction in $ZrTe_5$).

To obtain the LL spectrum, we perform the standard Peierls substitution using the Landau-gauge vector potential $\mathbf{A} = (-By, 0, 0)$. Following our previous works [6, 7], we implement the ladder operators and make the ansatz that the wavefunction has a form of

$$\psi_\uparrow = (c_1\phi_n, c_2\phi_{n-1}, c_3\phi_{n-1}, c_4\phi_n).$$

Here, ϕ_n is the eigenfunction of harmonic oscillator, n denotes the LL index, and $c_{1,2,3,4}$ are the coefficients to be determined. The effective Hamiltonian (1) now becomes

$$H = \begin{pmatrix} L_n - Z_o & \Delta\sqrt{n} & 0 & A_z k_z \\ \Delta\sqrt{n} & -L_n - Z_o & A_z k_z & 0 \\ 0 & A_z k_z & L_n + Z_o & -\Delta\sqrt{n} \\ A_z k_z & 0 & -\Delta\sqrt{n} & -L_n + Z_o \end{pmatrix} \tag{2}$$

with $L_n = M - n\mathscr{B}_\perp k_B^2 - \mathscr{B}_z k_z^2$, $k_B = \sqrt{2eB/\hbar}$, $\Delta = \sqrt{2e\hbar v_{F,\perp}^2 B}$, and $Z_o = e\mathscr{B}_\perp B/\hbar$. The eigenvalues (LLs) and eigenfunctions of this Hamiltonian do not have an analytical form except at $k_z = 0$ [7]. Therefore, a full LL spectrum with k_z dispersion (such as that in Fig. 1(b) of the main text) requires a numerical solution.

Fortunately, we can qualitatively describe the LL splitting from the above Hamiltonian. As one can see, the Z_o term in (2) behaves like the Zeeman effect, which breaks the degeneracy of each LL. But, its sign depends on the orbital character instead of the spin. Thus,

we can rewrite $Z_o = \frac{1}{2}g_o\mu_B B$, where μ_B is the Bohr magneton, and $g_o = 2e\mathscr{B}_\perp/\hbar\mu_B$ is the orbital g-factor. For $ZrTe_5$, given $\mathscr{B}_\perp = 0.1$ eV nm^2, $g_o = 5.2$.

To include the Zeeman effect, we employ the spin g-factor g_s and add a $Z_s = \frac{1}{2}g_s\mu_B B\sigma_z$ term to the effective Hamiltonian. It is instructive to look at the $n = 0$ LL on how the Zeeman effect would change the band inversion. For the $n = 0$ LL, Hamiltonian (2) reduces to

$$H = \begin{pmatrix} L_n - \frac{1}{2}g_{eff}\mu_B B & A_z k_z \\ A_z k_z & -L_n + \frac{1}{2}g_{eff}\mu_B B \end{pmatrix},$$

where $g_{eff} = g_o - g_s$ is an effective g-factor, leading to LL energy

$$E_0^\pm = \pm\sqrt{A_z^2 k_z^2 + (M - \mathscr{B}_z k_z^2 - \frac{1}{2}g_{eff}\mu_B B)^2}.$$

Following the analysis in the main text, we can define a field-dependent gap $\tilde{M}(B) = M - \frac{1}{2}g_{eff}\mu_B B$. For an inverted band structure ($g_o > 0$), when $g_{eff} > 0$, the application of the magnetic field could close the inverted gap ($\tilde{M}(B) \to 0$) and then reopen to form a normal gap. In our case, however, we find that the position of the anomalous $n = 0$ peak in the extreme quantum limit is sensitive to the sign and magnitude of g_{eff}. $g_s \approx 20 > g_o$ best describes our data with the $n = 0$ peak in magneto-thermopower centering at around 22 T. As a result, the electronic structure of our sample remains inverted for all magnetic fields.

3. Four-fold splitting in the n = 1 LL

As discussed in the main text, there are two possible assignments to the four-fold splitting in the $n = 1$ LL, depending on the splitting energy of the upper and lower sub-LLs (Δ_s) as compared to that of the Γ and ζ point splitting ($\Delta_{\Gamma\zeta}$). $\Delta_s > \Delta_{\Gamma\zeta}$ for assignment (1) while $\Delta_s < \Delta_{\Gamma\zeta}$ for assignment (2). The band alignment and peak assignments of these two cases are summarized in Fig. 6.

4. Magneto-resistance and magneto-thermopower calculation

The resistivity ρ_{xx} and thermopower S_{xx} can be calculated using the following relations,

$$\rho_{xx} = \frac{\sigma_{xx}}{\sigma_{xx}^2 + \sigma_{xy}^2},$$
$$S_{xx} = -\frac{\pi^2 k_B^2 T}{3e} \frac{1}{\sigma_{xx}^2 + \sigma_{xy}^2} \left(\sigma_{xx} \frac{\partial \sigma_{xx}}{\partial E_F} + \sigma_{xy} \frac{\partial \sigma_{xy}}{\partial E_F} \right),$$

where σ_{xx} (σ_{xy}) is the longitudinal (Hall) conductivity, and E_F is the Fermi energy. Following Refs. [4, 8], we can deduce the conductivities and their energy derivatives as

$$\sigma_{xx} = \frac{\hbar e^2}{2\pi^3 l_B^2} \int_0^{+\infty} dk_z \sum_{n,m} \frac{\langle m| v_x |n\rangle \langle n| v_x |m\rangle}{(E_F - E_n)^2 + \gamma^2} \frac{\gamma^2}{(E_F - E_m)^2 + \gamma^2},$$
$$\sigma_{xy} = i \frac{e^2 \hbar}{2\pi^2 l_B^2} \int_0^{+\infty} dk_z \sum_{n,m} \langle m| v_x |n\rangle \langle n| v_y |m\rangle \frac{f(E_m) - f(E_n)}{(E_m - E_n)^2},$$
$$\frac{d\sigma_{xx}}{dE_F} = -\frac{\hbar e^2}{\pi^3 l_B^2} \int_0^{+\infty} dk_z \sum_{n,m} \frac{\langle m| v_x |n\rangle \langle n| v_x |m\rangle}{(E_F - E_n)^2 + \gamma^2} \frac{\gamma^2}{(E_F - E_m)^2 + \gamma^2} \times \left[\frac{(E_F - E_m)}{(E_F - E_m)^2 + \gamma^2} + \frac{(E_F - E_n)}{(E_F - E_n)^2 + \gamma^2} \right],$$
$$\frac{d\sigma_{xy}}{dE_F} = i \frac{e^2 \hbar}{2\pi^2 l_B^2} \int_0^{+\infty} dk_z \sum_{n,m} \langle m| v_x |n\rangle \langle n| v_y |m\rangle \frac{1}{(E_m - E_n)^2} \frac{\partial}{\partial E_F} [f(E_m) - f(E_n)].$$

Here, $l_B = \sqrt{\hbar/eB}$ is the magnetic length, γ is the phenomenological broadening due to scattering, $f(E)$ is the Fermi distribution function, and m, n denote the eigenstates.

We use the following band parameters to calculate R_{xx} and S_{xx} in Fig. 4 of the main text: $\mathscr{B}_\perp = 0.1$ eV nm^2, $\mathscr{B}_z = 0.3$ eV nm^2, $v_{F,\perp} = 5 \times 10^5$ m/s, $v_{Fz} = 5 \times 10^4$ m/s, $M = 10$ meV, and $g_s = 20$. We set a constant scattering rate $\gamma = 0.6$ meV but allow for a small variation (within 10%) in $v_{F,\perp}$ between $k_\Gamma = 0$ and k_ζ points in accordance with the recent magneto-IR result [6]. As we do not have complete knowledge of the k_z dependence, the $v_{F,\perp}(k_z)$ relationship is relatively arbitrary and assumed to be

$$v_{F,\perp}(k_z) = v_{F,\perp} + a \left(\frac{E(k_z)}{M} - 1 \right) e^{-\frac{k_z}{k_c}}.$$

Here, $a = 1 \times 10^5$ m/s, $k_c = 2$ nm^{-1} is the cut-off wave vector, and $E(k_z)$ is the zero-field energy dispersion along the k_z direction described by Eq. (3) of the main text.

Finally, we can determine the magnetic field dependence of the Fermi energy self-

consistently through

$$N = \frac{1}{2\pi l_B^2} \int_0^{+\infty} \frac{dk_z}{\pi} \left[\sum_{m\in C} f(E_m) - \sum_{n\in V} (1 - f(E_n)) \right],$$

where the letter C (V) indicates the summation over the conduction (valence) energy levels, and N is the total carrier density. In our calculation, we use $N = 4.5 \times 10^{17}$ cm^{-3}. The DOS can then be calculated accordingly.

Overall, the model calculation of Fig. 4 is in qualitative agreement with the experimental results of Figs. 2 and 3 of the main text.

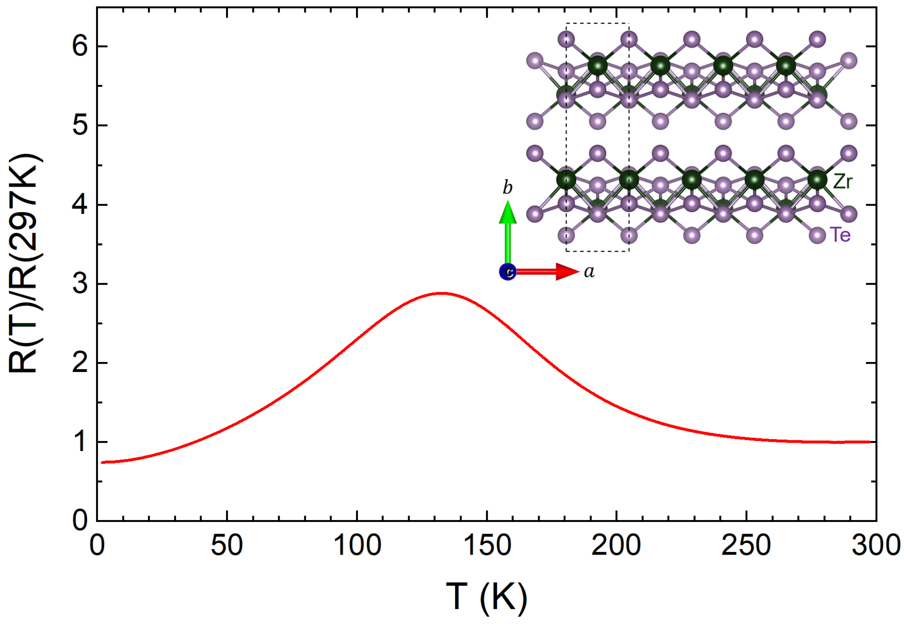

Fig. 1. R versus T curve of our $ZrTe_5$ sample. Normalized resistance, $R(T)/R(297\text{K})$, as a function of temperature measured along the a direction. Inset: Atomic structure of $ZrTe_5$ along the c axis. The dash line indicates a unit cell.

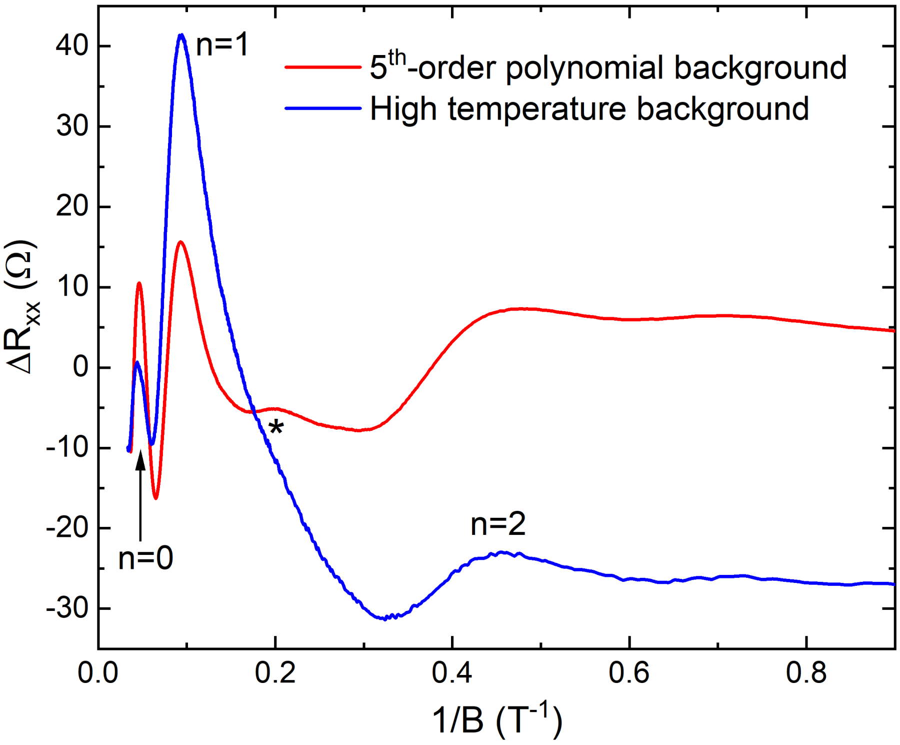

Fig. 2. Robust peak positions in ΔR_{xx} independent of background subtraction methods. Comparison of the oscillatory components in R_{xx} obtained by subtracting a smooth high-order polynomial (red) background and a high temperature (blue) background. The $\star$ symbol indicates a possible splitting of the $n = 1$ peak.

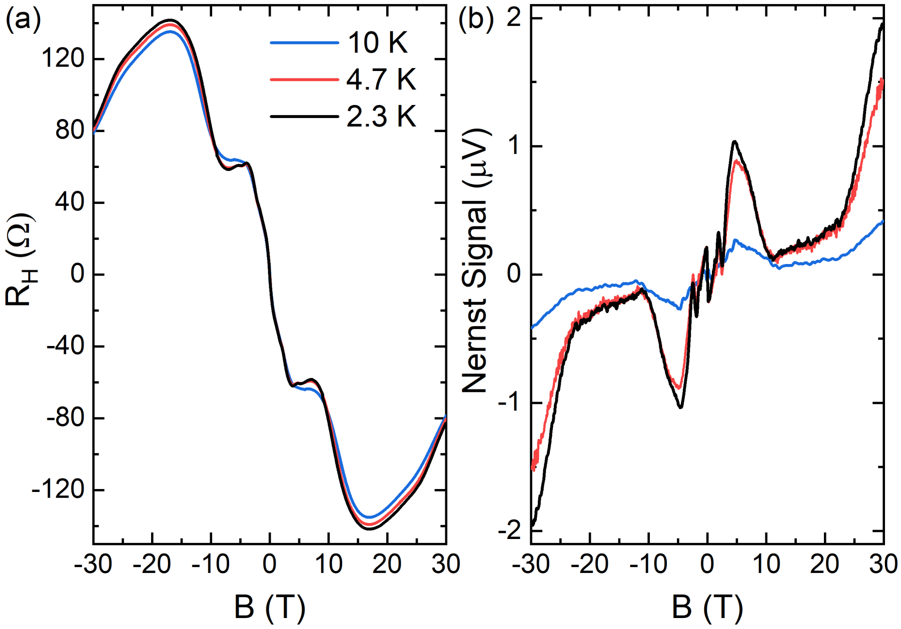

Fig. 3. Hall resistance (R_H, a) and Nernst signal (b) measured at low temperatures.

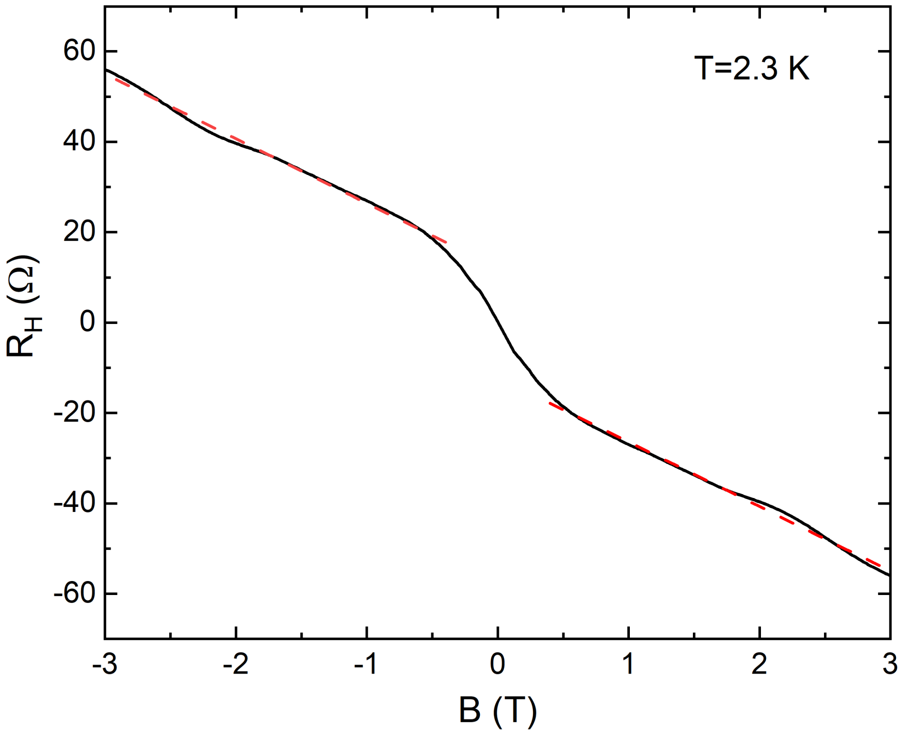

Fig. 4. Zoom-in view of the Hall resistance (R_H, black) data near zero magnetic field. The red dash lines indicate a linear fit to the higher field data by ignoring variations concomitant with the quantum oscillations.

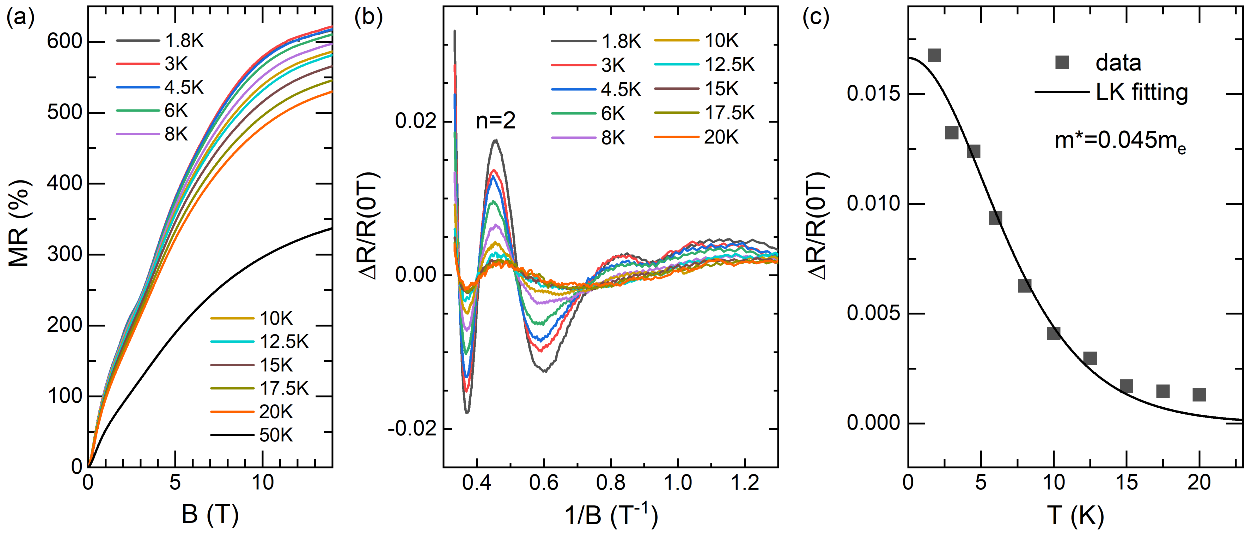

Fig. 5. Temperature-dependent MR measurements. (a) MR of $ZrTe_5$, measured at different temperatures. (b) Quantum oscillations in $\Delta R/R(0\mathrm{T})$ as a function of $1/B$ at different temperatures, obtained by subtracting a smooth background (we choose $R(50\mathrm{K})$ as the background) from R. (c) Oscillation amplitude of the $n = 2$ peak as a function of temperature. The solid line represents the best LK fitting, giving rise to an effective mass of $m^* = 0.045m_e$.

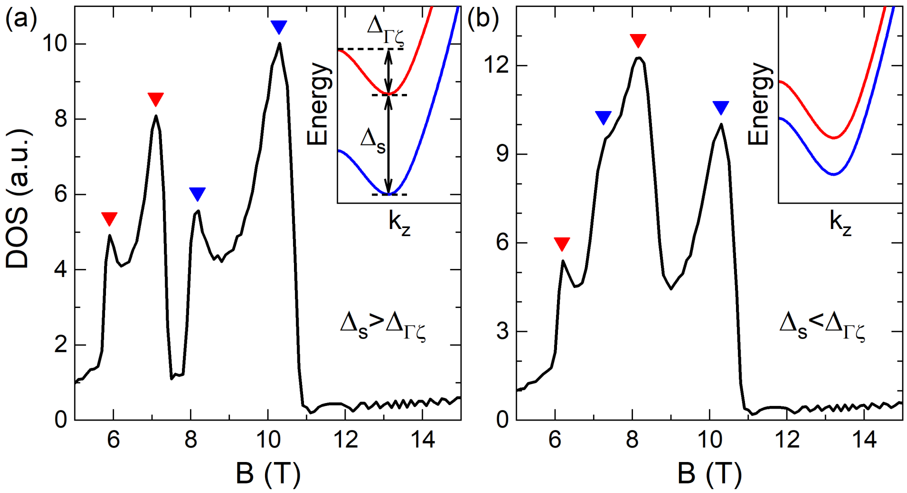

Fig. 6. Two possible assignments to the four-fold splitting in the $n = 1$ LL. The assignment in (a) corresponds to the case when the sub-LL splitting is larger than the Γ and ζ point splitting $\Delta_s > \Delta_{\Gamma\zeta}$, while (b) shows the opposite case $\Delta_s < \Delta_{\Gamma\zeta}$. The insets illustrate the band alignment in the two cases, with red and blue representing the upper and lower sub-LLs. Within the same color code, the lower (higher) magnetic field down-triangle represents the Γ (ζ) point, respectively.

* yuxuan.jiang@ahu.edu.cn

† zhigang.jiang@physics.gatech.edu

[1] Yu, W., Jiang, Y., Yang, J., Dun, Z. L., Zhou, H. D., Jiang, Z., Lu, P. & Pan, W. Quantum oscillations at integer and fractional Landau level indices in single-crystalline $ZrTe_5$. *Sci. Rep.* **6**, 35357 (2016).

[2] Liang, T., Lin, J., Gibson, Q., Kushwaha, S., Liu, M., Wang, W., Xiong, H., Sobota, J. A., Hashimoto, M., Kirchmann, P. S., Shen, Z.-X., Cava, R. J. & Ong, N. P. Anomalous Hall effect in $ZrTe_5$ *Nat. Phys.* **14**, 451-455 (2018).

[3] Sun, Z., Cao, Z., Cui, J., Zhu, C., Ma, D., Wang, H., Zhuo, W., Cheng, Z., Wang, Z., Wan, X. & Chen, X. H. Large Zeeman splitting induced anomalous Hall effect in $ZrTe_5$. *NPG Quantum Mater.* **5**, 36 (2020).

[4] Zhang, J. L., Wang, C. M., Guo, C. Y., Zhu, X. D., Zhang, Y., Yang, J. Y., Wang, Y. Q., Qu, Z., Pi, L., Lu, H.-Z. & Tian, M. L. Anomalous thermoelectric effects of $ZrTe_5$ in and beyond the quantum limit. *Phys. Rev. Lett.* **123**, 196602 (2019).

[5] Wang, J., Niu, J., Yan, B., Li, X., Bi, R., Yao, Y., Yu, D. & Wu, X. S. Vanishing quantum oscillations in Dirac semimetal $ZrTe_5$. *Proc, Natl. Acad. Sci.* **115**, 9145-9150 (2018).

[6] Jiang, Y., Wang, J., Zhao, T., Dun, Z. L., Huang, Q., Wu, X. S., Mourigal, M., Zhou, H. D., Pan, W., Ozerov, M., Smirnov, D. & Jiang, Z. Unraveling the topological phase of $ZrTe_5$ via magnetoinfrared spectroscopy. *Phys. Rev. Lett.* **125**, 046403 (2020).

[7] Jiang, Y., Dun, Z. L., Zhou, H. D., Lu, Z., Chen, K.-W., Moon, S., Besara, T., Siegrist, T. M., Baumbach, R. E., Smirnov, D. & Jiang, Z. Landau-level spectroscopy of massive Dirac fermions in single-crystalline $ZrTe_5$ thin flakes. *Phys. Rev. B* **96**, 041101(R) (2017).

[8] Wang, C. M., Lu, H.-Z. & Shen, S.-Q. Anomalous phase shift of quantum oscillations in 3D topological semimetals. *Phys. Rev. Lett.* **117**, 077201 (2016).
